# Sciatica Caused by Nerve Root Entrapment Due to a Prominent Lumbosacral Ligament

**DOI:** 10.5334/jbr-btr.840

**Published:** 2015-09-15

**Authors:** J. Gossner

**Affiliations:** 1 Department of Clinical Radiology, Evangelisches Krankenhaus Göttingen-Weende, Göttingen, Germany

A 61-year-old female patient presented at our hospital with worsening sciatica of the right leg. Examination revealed involvement of the fifth lumbar nerve root. Pain was the leading complaint. A computed tomographic scan of the lumbar spine was performed. At the disc level L5-S1 osteochondrosis and spondylarthrosis could be seen. There was no disc prolapse or formainal stenosis affecting the L5 root. A prominent lumbosacral ligament was present on both sides on computed tomography as well as on MRI (Fig. A, arrows). Coronal reformations revealed direct contact of the lumbosacral ligament and the nerve root (Figs. B, C). In contrast, there is no obvious contact on the left (Fig. C). The right nerve root appeared slightly edematous compared to the left side and the surrounding fat tissue seemed minimally infiltrated (Fig. A). A possible entrapment of the fifth nerve root on the right side was suggested. To test this hypothesis, a CT guided selective nerve root infiltration (local anesthetic and corticoid) was performed on the next day resulting in improvement of the patient’s pain (Fig. D). With further infiltrations the patient experienced a significant and lasting pain reduction.

**Figures A–D:**
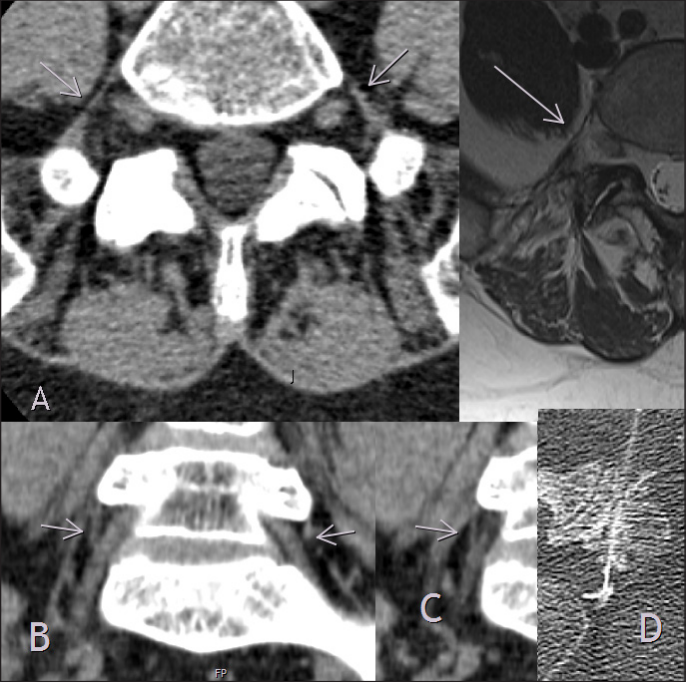


## Comment

The lumbosacral ligament is a fibrous structure reaching from the caudal parts of the transverse process and the body of the fifth lumbar vertebrae to the ala of the sacral bone. Briggs and Chandraraj observed in an anatomical study, that in some cases the lumbosacral ligament showed a contact to the underlying nerve root. In 9% of the dissected patients the nerve root was compressed and visibly flattened. Microscopic examination of these nerves showed an increase in connective tissues, suggestive of chronic changes due to entrapment. In the patients with compressed nerve roots the lumbosacral ligament was also prominent, presumably due to a reactive hypertrophy because of the degenerative disc disease and subsequent segmental microinstability. This cadaveric study suggested a possible role of the lumbosacral ligament in some patients with sciatica. But, until now, there have been no clinical studies or case reports on this subject. In our case the above described morphologic features of symptomatic entrapment were visible on the CT scan and CT guided infiltration of the affected fifth lumbar nerve root resulted in improvement of the patient sciatica. The combination of morphologic findings and improvement after selective nerve root infiltration favors the hypothesis of a symptomatic entrapment of the fifth lumbar nerve root due to a prominent lumbosacral ligament as a rare cause of sciatica. Radiologist should be aware of this possible differential diagnosis.

## Competing Interests

The author declares that they have no competing interests.
